# Technology-Enabled Self-Management of Chronic Obstructive Pulmonary Disease With or Without Asynchronous Remote Monitoring: Randomized Controlled Trial

**DOI:** 10.2196/18598

**Published:** 2020-07-30

**Authors:** Vess Stamenova, Kyle Liang, Rebecca Yang, Katrina Engel, Florence van Lieshout, Elizabeth Lalingo, Angelica Cheung, Adam Erwood, Maria Radina, Allen Greenwald, Payal Agarwal, Aman Sidhu, R Sacha Bhatia, James Shaw, Roshan Shafai, Onil Bhattacharyya

**Affiliations:** 1 Institute for Health System Solutions and Virtual Care Women's College Hospital Toronto, ON Canada; 2 Respiratory Therapy Department Markham Stouffville Hospital Markham, ON Canada; 3 Medicine, Care Transitions, Access & Flow, Respiratory Therapy Markham Stouffville Hospital Markham, ON Canada; 4 Support Services & Transformation Markham Stouffville Hospital Markham, ON Canada; 5 Center for Respiratory Health Markham Stouffville Hospital Markham, ON Canada; 6 Department of Family and Community Medicine University of Toronto Toronto, ON Canada; 7 University Health Network Toronto, ON Canada; 8 Department of Medicine University of Toronto Toronto, ON Canada; 9 Women’s College Research Institute Toronto, ON Canada; 10 Hospital to Home and Community Medicine Clinic Markham Stouffville Hospital Markham, ON Canada

**Keywords:** COPD, eHealth, telemedicine, remote consultation, self-care

## Abstract

**Background:**

Chronic obstructive pulmonary disease (COPD) is a leading cause of mortality and leads to frequent hospital admissions and emergency department (ED) visits. COPD exacerbations are an important patient outcome, and reducing their frequency would result in significant cost savings. Remote monitoring and self-monitoring could both help patients manage their symptoms and reduce the frequency of exacerbations, but they have different resource implications and have not been directly compared.

**Objective:**

This study aims to compare the effectiveness of implementing a technology-enabled self-monitoring program versus a technology-enabled remote monitoring program in patients with COPD compared with a standard care group.

**Methods:**

We conducted a 3-arm randomized controlled trial evaluating the effectiveness of a remote monitoring and a self-monitoring program relative to standard care. Patients with COPD were recruited from outpatient clinics and a pulmonary rehabilitation program. Patients in both interventions used a Bluetooth-enabled device kit to monitor oxygen saturation, blood pressure, temperature, weight, and symptoms, but only patients in the remote monitoring group were monitored by a respiratory therapist. All patients were assessed at baseline and at 3 and 6 months after program initiation. Outcomes included self-management skills, as measured by the Partners in Health (PIH) Scale; patient symptoms measured with the St George’s Respiratory Questionnaire (SGRQ); and the Bristol COPD Knowledge Questionnaire (BCKQ). Patients were also asked to self-report on health system use, and data on health use were collected from the hospital.

**Results:**

A total of 122 patients participated in the study: 40 in the standard care, 41 in the self-monitoring, and 41 in the remote monitoring groups. Although all 3 groups improved in PIH scores, BCKQ scores, and SGRQ impact scores, there were no significant differences among any of the groups. No effects were observed on the SGRQ activity or symptom scores or on hospitalizations, ED visits, or clinic visits.

**Conclusions:**

Despite regular use of the technology, patients with COPD assigned to remote monitoring or self-monitoring did not have any improvement in patient outcomes such as self-management skills, knowledge, or symptoms, or in health care use compared with each other or with a standard care group. This may be owing to low health care use at baseline, the lack of structured educational components in the intervention groups, and the lack of integration of the action plan with the technology.

**Trial Registration:**

ClinicalTrials.gov NCT03741855; https://clinicaltrials.gov/ct2/show/ NCT03741855

## Introduction

### Background

Chronic obstructive pulmonary disease (COPD) is the third leading cause of mortality worldwide [1], with 65 million people having moderate to severe COPD worldwide [2]. In Ontario, Canada, COPD accounts for 24% of hospital admissions and 24% of emergency department (ED) visits and is responsible for the highest percentage (18.8%) of 30-day ED readmissions [3]. Reducing the frequency of COPD exacerbations is an important patient outcome and could result in significant cost savings.

One approach to reducing exacerbations is to provide regular remote monitoring of patients from their homes. Remote monitoring requires patients to take measurements of their vital signs (oxygen, blood pressure, and symptoms) and to record them manually on paper [4] or to transmit them with devices using a phone or an internet line [5-7]. Recent developments such as Bluetooth technology, cloud-based storage, and Wi-Fi–enabled tablets have allowed data from remote devices to be uploaded automatically to a database accessible to patients, caregivers, and health care providers, either periodically or on an *as-needed* basis [8-10]. Remote monitoring programs are always monitored by a health care provider, even though they are sometimes referred to as *self-management* programs, as the recordings are taken by the patients [10]. Some remote monitoring programs also often have an educational component [11,12] such as coaching sessions to support self-management. COPD self-management behaviors include self-recognition and self-treatment of exacerbations (eg, taking medications); coping with breathlessness; and lifestyle changes such as quitting smoking, eating healthy, and exercising [13]. Self-management COPD interventions have generally been shown to be effective in improving quality of life measures [13,14], but a recent meta-analysis failed to show significant improvements in quality of life [15].

There is a large body of literature on COPD reporting on the effects of remote monitoring on patient outcomes and health care utilization, and several recent reviews have summarized these findings [16-18]. For example, Kruse et al [16] reported that the number of articles stating that patient outcomes improved overall with telemonitoring was approximately equal to that showing no improvement. Another review [17] reported that remote monitoring decreased ED admissions and hospitalizations but failed to impact other patient outcomes (mortality, outpatient visits, and length of stay). Hong and Lee [17] suggested that *integrated remote monitoring* programs (those that have educational components) may be more effective, especially when they target patients with more advanced diseases.

Educational components come at an additional cost to these programs [19] and even the simple act of monitoring patients remotely and connecting with them only when alerts are received requires dedicated staff. Few studies have looked at the effectiveness of *self-monitoring* programs. Self-monitoring programs ask patients to take their readings and receive automated feedback based on these readings without being actively monitored by a health provider [4,20]. Results from the few studies available in the literature have shown some promise in improving patient outcomes but they were feasibility trials that required larger samples and control group designs. A self-monitoring program, if noninferior to a remote monitoring program, would provide the opportunity for significant cost savings without compromising on patient outcomes. To our knowledge, no studies to date have directly compared a remote monitoring program with a self-monitoring program for patients with COPD.

### Objectives

The objective of our study was to compare the effectiveness of implementing a technology-enabled self-monitoring program versus a technology-enabled self- and remote-monitoring program (or simply remote monitoring) in a population of patients with COPD, compared with a standard care group. We hypothesized that both intervention programs would lead to improvements in self-management skills and respiratory symptoms relative to the standard care program. In addition, the technology-enabled remote monitoring programs may be more effective at increasing COPD knowledge than self-monitoring alone.

## Methods

### Study Setting

The study was conducted at a 309-bed community-based hospital in Ontario. Recruitment took place in a hospital-based outpatient COPD clinic, from the private practice of respirologists affiliated with the hospital, and from an outpatient COPD rehabilitation program.

### Trial Design

We conducted an open-label randomized controlled trial (RCT) comparing 2 technology-enabled interventions, a self-monitoring group and a remote monitoring group, relative to standard care. Patients were randomized in a 1:1:1 ratio to 1 of the 3 groups. The study recruitment started in April 2018 and was completed in September 2019. A full description of the protocol has been published [21]. We report here only on the quantitative portion of the evaluation. The qualitative results are published separately [22].

### Participants

#### Eligibility Criteria

Patients were included if they were aged 18 years or older and had an established clinical diagnosis of COPD by a respirologist, according to clinical guidelines [23]. Exclusion criteria included a diagnosis of other significant lung diseases (eg, interstitial lung disease), patients without Wi-Fi internet access in their homes, inability to read English (required for filling out the questionnaires), participation in other remote monitoring programs, or inability to use the technology because of physical or cognitive impairment.

#### Recruitment Process

The main site of recruitment was the hospital-based outpatient clinic, where all eligible patients seen within the past year were contacted for participation. Patients could also be referred to the study from outside the clinic, through the private practice of hospital-affiliated respirologists or through an outpatient COPD rehabilitation program. Patients were contacted by phone, directly approached at an appointment, or approached at the hospital’s exercise rehabilitation program by a clinical staff member (respirologist or respiratory therapist [RT]). Those who were interested were referred to the clinical project specialist and scheduled for a baseline evaluation, at which time informed consent was obtained, group allocation was revealed, and the kit was provided (if in the self-monitoring or remote monitoring group).

#### Allocation

We used a web-based random number generator [24] to allocate patients to groups, as described in the trial protocol [21]. Sequential patient group allocation was placed in a sealed envelope and revealed to the patient by the clinical project specialist after consent was obtained.

### Intervention

#### Technology

The *Cloud DX Connected Health Kit* (Cloud DX Inc) [25] (Multimedia Appendix 1) was used in the 2 intervention groups. It was selected as it was made by a local Ontario company (a requirement from the granting agency), was fully developed, was on the market at the time of the study, and was capable of monitoring oxygen saturation. The kit comprised the following Bluetooth devices: a custom tablet computer, a Pulsewave wrist cuff monitor (which measures blood pressure), an oximeter, a weighing scale, and a thermometer. The devices were approved by the US Food and Drug Administration and Health Canada. A digital version of the COPD Assessment Test (CAT) [26] and the modified Medical Research Council (MRC) Scale [27] were also embedded in the technology. The data from all devices were transmitted to a database, and patients and health care providers interacted with it through a web-based portal. Regular bug fixes were occurring throughout the trial, and no major revisions of the content of the platform were done. Throughout the trial, there were 3 releases and 1 service pack installed in the platform and 2 releases, 5 hotfixes, and 1 service pack released for the companion app (see Multimedia Appendix 2 for details).

#### Intervention Procedures

The intervention lasted for 6 months. Patients in the intervention groups were asked to record their vitals (oximetry and blood pressure were required, whereas temperature and weight were optional) and symptoms (CAT and MRC) with the Cloud DX platform every day. They were also provided with a written version of a personalized COPD action plan that instructed patients on what to do if their readings fell outside predetermined thresholds (Multimedia Appendix 1). Individual patient thresholds were determined by the clinical project specialist (who was an RT), in consultation with the patient’s respirologist. Patients in the self-monitoring and remote monitoring groups were additionally contacted by the clinical project specialist 2 weeks after receiving their kit to reassess the appropriateness of the thresholds. In addition, all patients had the option to email or call the clinic with any nonemergency questions they may have. All patients were advised to go to the ED if necessary, at any point in the study. Patients were also informed that data were not monitored 24 hours, 7 days a week and to respond to their clinical needs as they would normally do outside of the study.

When a patient’s readings fell outside the predetermined thresholds, a notification was sent to both the clinical project specialist and the patient through email. The clinical project specialist reviewed the readings and responded when clinically indicated only for the remote monitoring group. Follow-up calls were made only when the readings exceeded thresholds twice or more within 2 days and were made only on weekdays. An attempt to complete the follow-up call was performed within 24 hours of receiving the notification. If the patient was unavailable, a message was left to return the call. In addition, the RT called the patients in the remote monitoring group once a week, irrespective of the values of the vitals. The purpose of the call was to check the patients, prompt action plan usage as needed, and provide education to the patients about their COPD as needed. The clinical project specialist received the readings for the self-monitoring group, but they were not actively monitored, and no follow-up calls were made in this group. Patients in the self-monitoring group were informed that their data were not actively monitored by the clinic. Patients in both intervention groups had secondary threshold levels (extreme measures) preset by the site investigator. Cloud DX staff monitored these levels and contacted the patients when necessary. For details, please refer to the protocol [21].

Patients in the standard care group were not provided with a technology or an action plan. This group received otherwise standard care from the respiratory clinic, including routine in-person follow-up appointments and access to a certified respiratory educator. Patients in the standard care group were told that they would receive the equipment at the end of the trial to incentivize them to stay in the trial and to ensure that all participants had equal access.

### Outcomes

All patients completed 3 assessments, at baseline, at 3 months, and at 6 months, on a series of questionnaires. Visit 1 (baseline) was in person, whereas visits 2 (3 months) and 3 (6 months) could be done in person or remotely (online through REDCap [28,29] or over the phone).

#### Primary Outcome

The primary outcome for the trial was self-management as assessed using the Partners in Health (PIH) Scale [30], a validated scale measuring the current status of self-management, with items on the knowledge of the condition and skills to monitor and respond to symptoms. This scale was selected to measure the primary outcome as we believed that both interventions could lead to self-management improvement.

#### Secondary Outcomes

The secondary outcomes included measures of COPD severity and COPD knowledge and were measured with the St George’s Respiratory Questionnaire (SGRQ) [31] and the Bristol COPD Knowledge Questionnaire (BCKQ) [32]. The SGRQ contains subsections on respiratory symptoms, activities that are limited because of breathlessness, and impacts on daily life. The BCKQ [32] is a measurement of the level of knowledge of the disease in patients with COPD. Patients were also asked to self-report at baseline, 3 months, and 6 months on their COPD-related ED presentations, hospital admissions, length of hospital stays, number of exacerbations (episodes in which antibiotics or steroids were prescribed or hospital/clinic visits because of a respiratory issue), number of COPD-related visits to a family doctor, number of COPD-related nurse contacts, self-reported use of medication, and self-reported smoking cessation. The number of contacts/calls to the outpatient clinic and deaths were tracked and reported by the clinical project specialist. In addition, hospital admission data and ED usage from the local hospital were also obtained.

Vendor-recorded use data were also documented and sent for analysis at the end of the trial. This included the frequency of recordings for oxygen, blood pressure, temperature, weight, MRC and CAT scores, and the number of times thresholds were exceeded.

### Statistical Analysis

Patient characteristics were summarized using descriptive statistics, including mean and SD for continuous variables (if normally distributed) or median, median absolute deviation, and absolute numbers for categorical variables.

All quantitative continuous data were analyzed by conducting a between-group repeated measures analysis of variance (ANOVA) comparing the scores at baseline versus 3-month follow-up and baseline versus 6-month follow-up assessments. This deviation from the original protocol (where we had planned to include all 3 time points in each analysis) was done to maximize the data and avoid excluding participants who did not have data on all 3 time points. Kruskal-Wallis tests were used where data were not normally distributed or group variances were heterogeneous.

### Ethics and Dissemination

The study was approved by the research ethics boards of the Markham Stouffville Hospital and Women’s College Hospital, Ontario, Canada (protocol version 1.8, December 7, 2018). The study was also retrospectively registered with ClinicalTrials.gov (NCT03741855).

### Patient and Public Involvement

During the initial planning stages of the study, we used a co-design approach in the development of the intervention. Patients were given access to the technology for 2 weeks and were subsequently interviewed about their experiences. Health care providers were also interviewed about their current models of care and their experience with the technology. The goal of this process was to establish whether the technology met the needs of its users (patients and health care providers) and to determine whether any modifications to the technology and the service it provided were needed. Modifications to both service and technology were done in response to this feedback. Some of this feedback was also used to inform the decisions about primary and secondary outcome selection.

Patient advisers were not involved directly in the development of the research question and outcome measures or recruitment. The burden of the intervention was assessed by the research ethics boards who had public member representatives. Any participants interested in receiving information about the results of the study will be provided with a summary once the results are available.

## Results

### Study Participants

**Figure 1:**
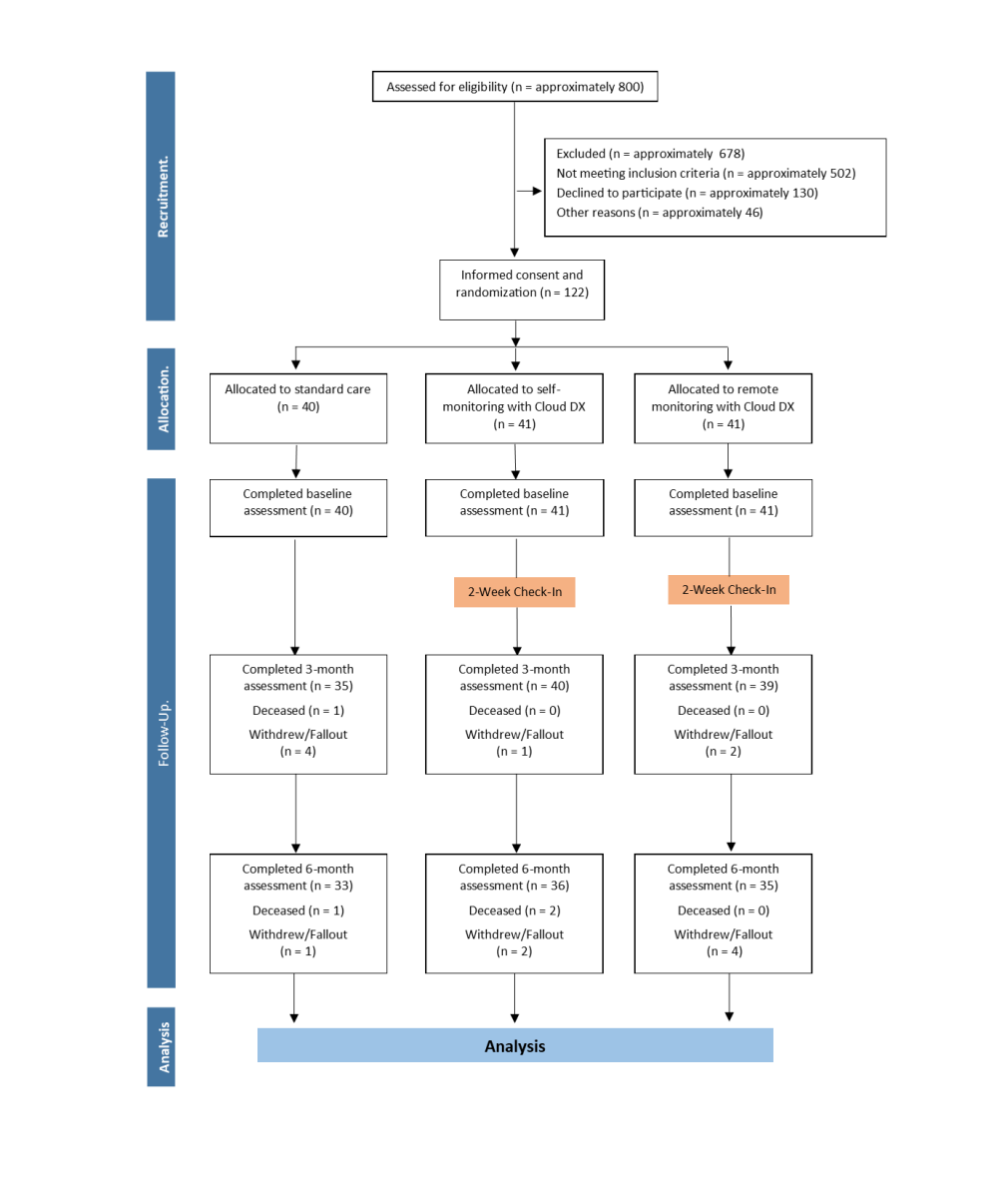
Patient flow through the study. Patient flow through each arm of the study. A total of approximately 800 patients were screened for eligibility in order to obtain the final sample of 122 participants.

A total of 122 patients participated in the study: 40 in the standard care, 41 in the self-monitoring, and 41 in the remote monitoring groups. Of these patients, 7 in the standard care, 5 in the self-monitoring, and 6 in the remote monitoring group did not complete the study (8 patients withdrew from the trial for various reasons; 6 patients were noncompliant with their readings; 4 patients died: 1 from a COPD exacerbation, 1 from complications of comorbid conditions, 1 from cardiac arrest, and 1 from unknown causes; and 1 patient dropped out because of difficulty using the technology; Figure 1). There were no significant differences in the rates of study completion among the groups (*P=*.80). Patients were excluded from individual analyses, if they had missing data.

The baseline characteristics of the patients are described in Table 1. Comparisons among groups were made with ANOVA for normally distributed variables, Kruskal-Wallis tests for variables that were not normally distributed, and chi-square tests for categorical data. Patients matched at baseline on all characteristics, except for the CAT baseline scores, where the self-monitoring group had significantly lower scores than the standard care group (*P=*.02).

All patients were able to speak and read English, except for 3 patients in the remote monitoring group who were included as they had support from caregivers in completing the questionnaires. There was an equal distribution of education level across the 3 groups (*P=*.64; Multimedia Appendix 1). Patients from all 3 groups were present in all income brackets, except for the highest income bracket, where there were no self-monitoring patients (*P=*.01; Multimedia Appendix 1). Patients in all 3 groups were also matched on a series of medical conditions at baseline (Multimedia Appendix 1), except for osteoporosis, for which the rates were lower in the remote monitoring group (*P*=.02), and pulmonary hypertension, which was reported only in 3 cases, all in the standard care group (*P*=.04).

**Table 1:** Baseline characteristics of patients in each group.

Characteristics		Standard care group	Self-monitoring group	Remote monitoring group	*P* value
**Age** **(years)**					.86
	Patients, n	40	41	41	
	Mean (SD)	72.78 (9.16)	71.76 (7.28)	71.98 (9.52)	
**Gender, n (%)**					.93
	Female	19 (48)	18 (44)	18 (44)	
	Male	21 (52)	23 (56)	23 (56)	
Patients with caregiver (%)		75	83	76	.63
**Years since diagnosis of COPD^a^**					.32
	Patients, n	36	40	38	
	Median	4	4.5	7	
	MAD^b^	3.71	5.19	4.45	
Currently smoking (%)		23	12	24	.32
Never smoked (%)		3	11	23	.07
**Years since quitting smoking**					.63
	Patients, n	29	31	24	
	Median	15	15	13	
	MAD	17.79	10.38	11.86	
**FEV1 % Pre^c^**					.22
	Patients, n	35	37	36	
	Median	0.45	0.53	0.50	
	MAD	0.22	0.13	0.25	
**FEV1 (L)^d^**					.09
	Patients, n	35	37	36	
	Median	1.09	1.26	1.17	
	MAD	0.47	0.67	0.43	
**FEV1/FVC^e^**					.49
	Patients, n	35	37	36	
	Median	0.56	0.58	0.54	
	MAD	0.18	0.16	0.19	
**COPD Assessment Test**					.02
	Patients, n	31	41	41	
	Mean (SD)	20.42 (7.68)	15.54 (7.65)	19.15 (8.18)	
**Systolic blood pressure**					.54
	Patients, n	25	41	40	
	Mean (SD)	129.36 (15.86)	129.90 (20.50)	125.65 (17.34)	
**Diastolic blood pressure**					.63
	Patients, n	25	41	40	
	Mean (SD)	76.76 (7.55)	75.37 (10.63)	74.48 (8.83)	
**BMI**					.08
	Patients, n	32	35	31	
	Median	24.65	28.10	23.40	
	MAD	6.67	4.15	6.52	
Never been in exercise/rehabilitation (%)		58	68	80	.08
Never used technology (%)		68	78	85	.16
No medications on hold (%)		63	59	63	.91
**COPD exacerbations in the past 12 months**					.17
	Patients, n	40	40	41	
	Median	1	1	2	
	MAD	1.48	1.48	2.97	
**Emergency department visits in the past 12 months**					.61
	Patients, n	40	41	41	
	Median	0	0	0	
	MAD	0	0	0	
**Hospitalizations in the past 12 months**					.72
	Patients, n	39	41	41	
	Median	0	0	0	
	MAD	0	0	0	
**Primary care visits in the past 12 months**					.79
	Patients, n	38	40	41	
	Median	1.5	1	1	
	MAD	2.22	1.48	1.48	

^a^COPD: chronic obstructive pulmonary disease.

^b^MAD: median absolute deviation.

^c^FEV1 % pre: % of predicted forced expiratory volume in 1 second.

^d^FEV1: forced expiratory volume in 1 second.

^e^FVC: forced vital capacity.

### Readings and Notifications

There were no significant differences in the number of readings completed by each intervention group on any of the measures. There were also no significant differences in the number of notifications received on any of the measures. Patients took their readings almost daily, with a median number of 160 readings in the self-monitoring group and 162 readings in the remote monitoring group over a 182-day period (Multimedia Appendix 1).

### Incoming Calls

There were differences among the groups in the number of incoming calls completed during the intervention (*P*<.001). Post hoc comparisons showed that the standard care group made significantly fewer calls (mean 0.13, SD 0.40) than the self-monitoring (mean 4.17, SD 4.17; *P*<.001) and the remote monitoring groups (mean 3.27, SD 4.29; *P*<.001). There was no difference between the number of calls made by the self-monitoring and remote monitoring groups (*P=*.11).

### Primary and Secondary Outcomes

**Figure 2:**
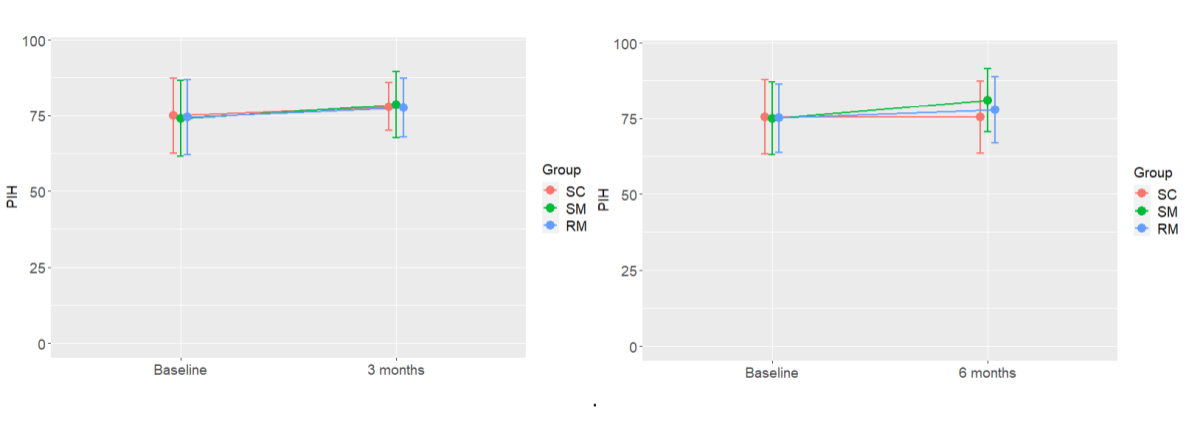
PIH at baseline and 3 months and baseline and 6 months for each group. Significant improvement in PIH scores from baseline to 3 months (*P*=.001) and from baseline to 6 months (*P*=.008) were observed, but no group effects or interactions, suggesting no differential effect among the groups. PIH: Partners in Health; RM: remote monitoring SC: standard care; SM: self-monitoring.

A repeated measures ANOVA showed a significant improvement in PIH scores from baseline to 3 months (*P=*.001) and from baseline to 6 months (*P=*.008) but no group effects or interactions, suggesting that there was no differential effect among the groups (Figure 2).

A repeated measures ANOVA showed a significant improvement in BCKQ scores from baseline to 3 months (*P*<.001) and from baseline to 6 months (*P*<.001; Multimedia Appendix 1). Steeper gains were observed in the remote monitoring group compared with the self-monitoring and standard care groups, both from baseline to 3 months and from baseline to 6 months, but the interaction effect did not reach statistical significance (*P=*.13 and *P=*.07, respectively). The gains in accuracy were less than 10%, and all groups had initial scores of just above 30% accuracy, which is lower than the average of 54% accuracy reported by the original BCKQ study [32]. No group main effects were observed.

A repeated measures ANOVA showed no changes in SGRQ activity scores (Multimedia Appendix 1) from baseline to 3 months (*P=*.49) or from baseline to 6 months (*P=*.76) and no group effects or interactions.

A repeated measures ANOVA showed a significant improvement in SGRQ impact scores (Multimedia Appendix 1) from baseline to 3 months (*P=*.047), but no significant group effect or interaction, suggesting that there was no differential effect among the groups. When comparing baseline to 6 months, a repeated measures ANOVA showed a significant effect of time (*P=*.006) and a significant interaction effect (*P=*.005). Separate pairwise comparison analyses were performed to examine the interaction effect. The standard care group improved from baseline to 6 months, whereas the remote monitoring group scores deteriorated (higher score) as demonstrated by a significant interaction effect (*P=*.02). A significant interaction effect (*P=*.003) was also observed when the self-monitoring and remote monitoring groups were analyzed separately, showing that the self-monitoring group improved, whereas the remote monitoring group worsened. Both standard care and self-monitoring groups improved significantly with time in their SGRQ impact scores (*P=*.002), and there was no interaction or group effect.

A repeated measures ANOVA showed no changes in SGRQ symptom scores from baseline to 3 months (*P=*.56) or from baseline to 6 months (*P=*.62) and no group effects or interactions (Multimedia Appendix 1).

Finally, for the remote monitoring and self-monitoring groups, a repeated measures ANOVA comparing the second CAT readings to their previous CAT reading, with time as a within-subject variable and group as a between-subject variable, showed no significant main effects or interactions. The same was observed with the MRC scores. Therefore, there were no changes in the CAT and MRC scores from the beginning of the intervention until the end.

### Correlation Between Changes in Partners in Health Scores and the Number of Readings

We ran a series of Pearson correlations between the number of readings (CAT, MRC, and oxygen saturation) and the change in score from baseline to 6 months for the participants in the self-monitoring and remote monitoring groups. No correlations were observed in any of these analyses.

### Health Care Use

In comparing baseline to 3 months, there were no significant effects of time, group, or interaction on any of the measures, except for a decrease in primary care COPD-related visits (*P=*.04). This reduction was most evident in the standard care and remote monitoring groups. In comparing baseline to 6 months, there were no effects of time, group, or interaction on any of the measures. The incidence of any of the above events was quite low (Multimedia Appendix 1).

In addition to the self-reported measures, the hospital charts of patients were reviewed to estimate the number of ED visits and hospitalizations that had occurred at the hospital during the 6 months preceding their enrollment and during their participation in the trial. The charts were also reviewed to assess the total number of clinic visits that the patients had completed during these periods (clinic visits could be ascertained only for patients who were seeing a physician at the COPD clinic). Nonparametric comparisons were run to estimate the effect of time, group, and time×group interaction. No significant main effects or interactions were observed.

Separate data analyses were performed to compare only COPD-related ED visits and hospitalizations. These analyses showed a significant decrease in COPD-related ED visits during the 6 months before trial enrollment to the 6 months during trial enrollment (*P=*.007); however, there was no group effect or interaction, suggesting that there was a general decline in visits across groups. For COPD-related hospital admissions, there was a decrease but not a statistically significant effect across the 3 groups (*P=*.07; Table 2).

**Table 2:** Health care use based on hospital data.

Group		Patients, n	Six months before enrollment				Six months post enrollment			
			Mean (SD)	Median	MAD^a^	Maximum value	Mean (SD)	Median	MAD	Maximum value
**Emergency department admissions**										
	SC^b^	40	0.7 (0.99)	0	0	3	0.43 (0.9)	0	0	4
	SM^c^	41	0.22 (0.52)	0	0	2	0.32 (0.79)	0	0	4
	RM^d^	41	0.46 (0.9)	0	0	4	0.37 (0.77)	0	0	4
**Hospital admissions**										
	SC	40	0.25 (0.54)	0	0	2	0.3 (0.85)	0	0	5
	SM	41	0.15 (0.48)	0	0	2	0.12 (0.4)	0	0	2
	RM	41	0.32 (0.82)	0	0	4	0.15 (0.42)	0	0	2
**Emergency department admissions for COPD^e^**										
	SC	40	0.38 (0.74)	0	0	3	0.13 (0.52)	0	0	3
	SM	41	0.15 (0.48)	0	0	2	0.07 (0.35)	0	0	2
	RM	41	0.27 (0.71)	0	0	4	0.1 (0.3)	0	0	1
**Hospital admissions for COPD**										
	SC	40	0.15 (0.43)	0	0	2	0.18 (0.81)	0	0	5
	SM	41	0.07 (0.35)	0	0	2	0.02 (0.16)	0	0	1
	RM	41	0.2 (0.56)	0	0	3	0.05 (0.22)	0	0	1
**Clinic visits**										
	SC	32	1.25 (1.08)	1	0	6	1.41 (0.98)	1	0	4
	SM	35	1.23 (0.97)	1	1.5	4	1.57 (0.78)	1	1.5	4
	RM	30	1.37 (0.72)	1	0	3	1.5 (0.97)	1	0.7	4

^a^MAD: median absolute deviation.

^b^SC: standard care.

^c^SM: self-monitoring.

^d^RM: remote monitoring.

^e^COPD: chronic obstructive pulmonary disease.

## Discussion

### Principal Findings

This study compared the effectiveness of a technology-enabled self-monitoring program to a remote monitoring program and standard care in a population of patients with COPD. Despite high adherence to the intervention and a low dropout rate, the study found no difference in self-efficacy or disease knowledge and disease severity measures among the groups. All 3 groups, including the standard care group, improved self-efficacy and disease knowledge measures. These changes were significant over time and were evident at both 3- and 6-month evaluations. The standard care and self-monitoring groups, but not the remote monitoring group, also reported a lower impact of COPD on their lives when comparing baseline to 6-month evaluations. There were no changes in symptoms or activity scores in any of the groups. There were also no differences (increases or decreases) in patient health care utilization, including ED visits, hospital admissions, primary care visits, or nursing visits, during participants’ participation in the trial relative to the 6 months preceding the trial, although these were secondary outcomes that the study was not powered for.

The lack of effect in this study is not unique, as current studies and reviews on the effects of remote monitoring on patient outcomes and health utilization have shown mixed results [16-18]. Some studies have reported positive results on some quality of life measures and symptoms [10,33-35], but many have reported no effects [8,12,36-39]. With respect to health care utilization, the effects are also mixed, with some reporting reductions in hospitalizations [5,7,33,40-42], length of stay [7,33,40,43], and ED visits [33,40], but many failing to find significant effects [6,10,34,36-38]. A recent systematic review concluded that the evidence on the effectiveness of remote monitoring is mixed [16], although some meta-analyses have reported significant reductions in hospitalizations and ED visits [17,44]. Hong and Lee [17] took their meta-analysis one step further and examined the effects of patient severity and intervention type (interventions with or without an educational component). They concluded that interventions with an educational component (such as those seen in self-management programs) and those targeting patients with more severe diseases had the greatest effects, especially on health care utilization (hospitalizations and ED visits). Our intervention lacked both these components.

First, although spirometry readings on an average suggested moderate to severe disease, there was a large variability among patients, with many patients having milder symptoms. Most patients had no hospital or ED admissions in the 12 months before joining the intervention, which also suggested that even those with moderate to severe disease had enough clinical support to avoid hospitalizations. However, we know that there was room for improvement as all 3 groups improved on several patient outcomes, including self-efficacy and disease knowledge measures. This may mean that the standard care delivered at the local clinic may already have been quite effective in improving patient outcomes, which would have made it harder for us to detect any additional effect of remote monitoring or self-monitoring over those provided in standard care during the same period. If future studies exclude milder patients from similar interventions and find effects, it may provide evidence that remote monitoring and self-monitoring monitoring programs are better suited for moderate to severe patients. This may also make such programs more affordable for health care systems as only a subset of patients will have to be monitored. If the primary goal is to examine the impact on health care utilization (which was not our focus in this study), we recommend focusing on patients with at least one ED or hospital admission, using administrative data sets and examining longer intervention periods.

Second, our intervention did not have a formal educational component. Although the clinical project specialist was making regular calls to patients in the remote monitoring group and providing them with guidance and education when needed, there was no structured educational component in the form of coaching sessions. Some education was also already delivered as part of standard care in the clinic, which may also explain why patients with moderate to severe disease had relatively few hospitalizations. Future studies should include structured coaching sessions, covering topics on self-recognition and self-treatment of exacerbations (eg, taking medications), coping with breathlessness, and lifestyle changes [13] to make the intervention groups more distinct from standard care. More personalized educational components can be delivered by a health care professional in a remote monitoring program, whereas more standardized education modules can be delivered directly through the device in response to readings.

In this intervention, we provided patients with action plans that were not integrated with the devices. Evidence suggests that action plans can be effective in reducing the effects of exacerbations when they are followed, but few patients follow written action plans [45]. The integration of action plans within the platform, with patients receiving feedback directly from the device, may improve their use, although a recent study found no additional benefits when action plans were embedded within a self-monitoring mobile app over those provided by a written action plan [38]. Further developments in trend analyses and predictive analytics of remote monitoring data [46] may allow for early detection of exacerbations as relative changes in vital signs may be more important than detecting vitals reaching absolute thresholds.

### Strengths and Limitations

With regard to strengths, this is the first study to directly compare remote monitoring relative to self-monitoring and standard care, and the comparison was conducted through an RCT, which offers strong internal validity for the findings. There was a high adherence rate from both intervention groups with hundreds of oximeter recordings, subjective symptom scores, and blood pressure measurements taken from patients. Furthermore, follow-up was very good, with only 7% of the participants withdrawing, 7% being noncompliant, and only one patient finding the technology to be too difficult to use. Despite this, our design suffered from drawbacks such as a relatively short intervention period (6 months) and inclusion criteria that allowed any patients with a diagnosis of COPD, irrespective of disease severity, to participate. Some of these decisions were made because of time constraints surrounding the trial funding and associated recruitment challenges. Many studies in the literature had disease severity inclusion criteria that often required patients to be admitted at least once and often twice in the previous year [8,42,47]. With respect to intervention duration, a full-year intervention seems to be the most common, but we noted interventions ranging from 3 to 24 months. Although our intervention was only 6 months, it is worth noting that some 6-month interventions have shown positive effects [7,34,35].

### Conclusions

Our 6-month intervention comparing technology-enabled remote monitoring and self-monitoring programs showed no intervention specific improvements in self-efficacy, disease knowledge, or quality of life. No effects were observed in health care utilization, including hospital admissions and ED visits. Future studies should focus on patients with higher health care system use and moderate to severe disease. We also recommend including structured educational components (potentially both in remote monitoring and self-monitoring programs) and predictive analytics of vitals data that detect relative rather than absolute changes in vitals.

## Appendix

Multimedia Appendix 1 Supplementary results.

Multimedia Appendix 2 Bug fixes and releases.

Multimedia Appendix 3 CONSORT-eHEALTH checklist (V 1.6.1).

## Abbreviations

ANOVA: analysis of variance
BCKQ: Bristol COPD Knowledge Questionnaire
CAT: COPD Assessment Test
COPD: chronic obstructive pulmonary disease
ED: emergency department
MRC: Medical Research Council
PIH: Partners in Health
RCT: randomized controlled trial
RT: respiratory therapist
SGRQ: St George’s Respiratory Questionnaire
