# Supplementary material for: Technology-Enabled Self-Management of Chronic Obstructive Pulmonary Disease With or Without Asynchronous Remote Monitoring: Randomized Controlled Trial
Source: J Med Internet Res. 2020 Jul 30;22(7):e18598. doi: 10.2196/18598 (PMC7426797; doi:10.2196/18598)
Supplement: Multimedia Appendix 1 [file jmir_v22i7e18598_app1.docx]

Figure 1. The Cloud DX Connected Health Kit [13] consisting of a custom tablet computer, a Pulsewave® wrist cuff monitor (which measures blood pressure), an oximeter, weight scale and thermometer.


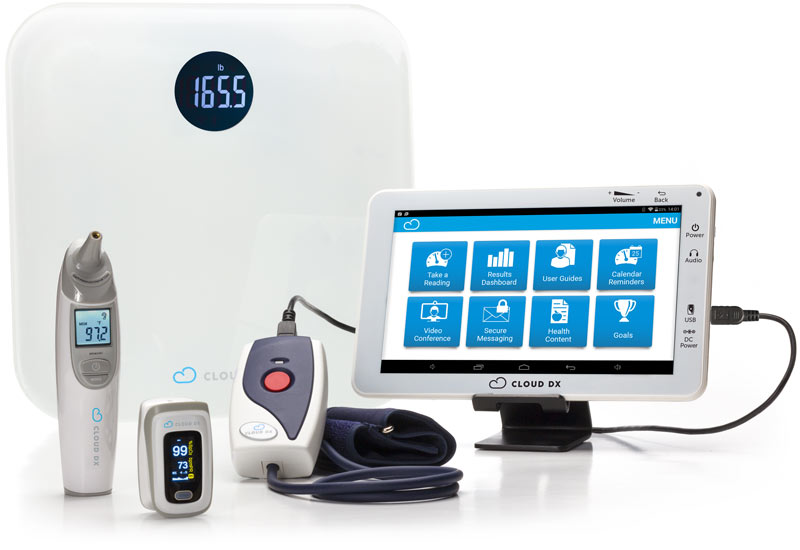


Figure 2. Action Plan. The action plan given to participants in the SM and RM groups. The action plan was provided on a piece of paper and patients were asked to refer to it when needed.


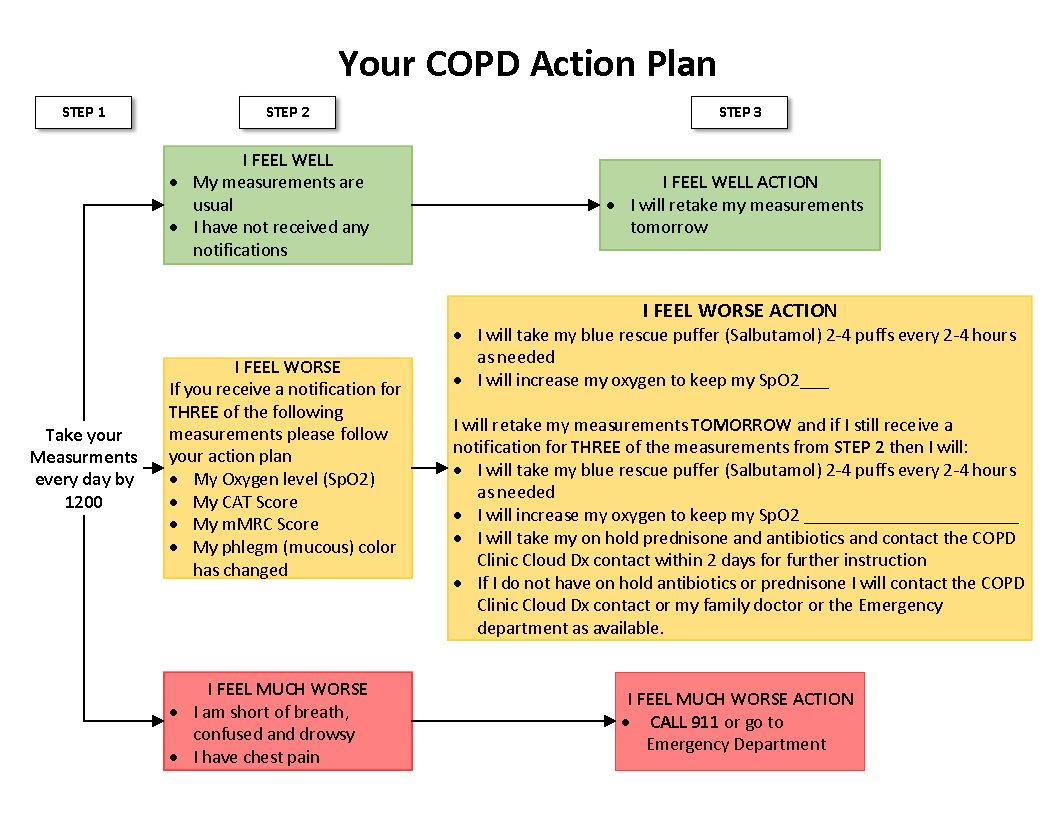


Figure 3. Education level of participants across groups.


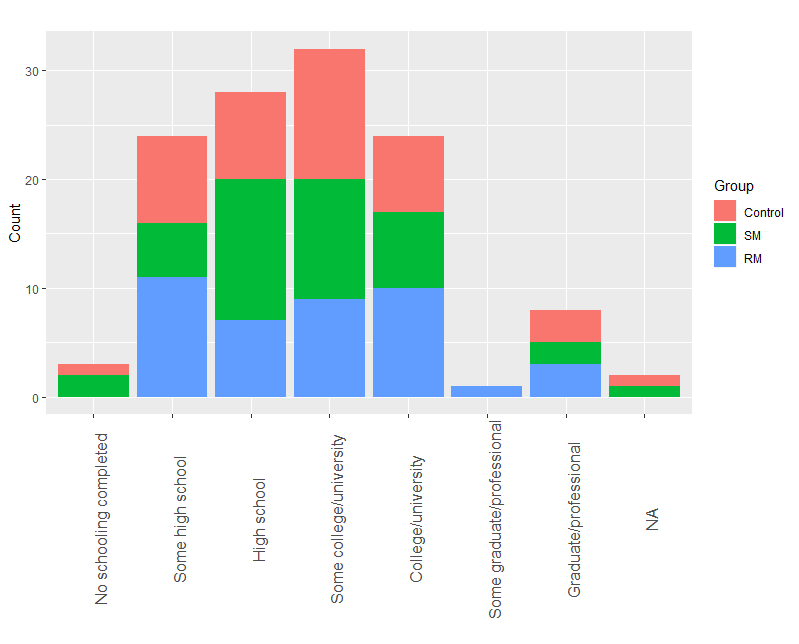


Figure 4. Income levels of participants across groups.


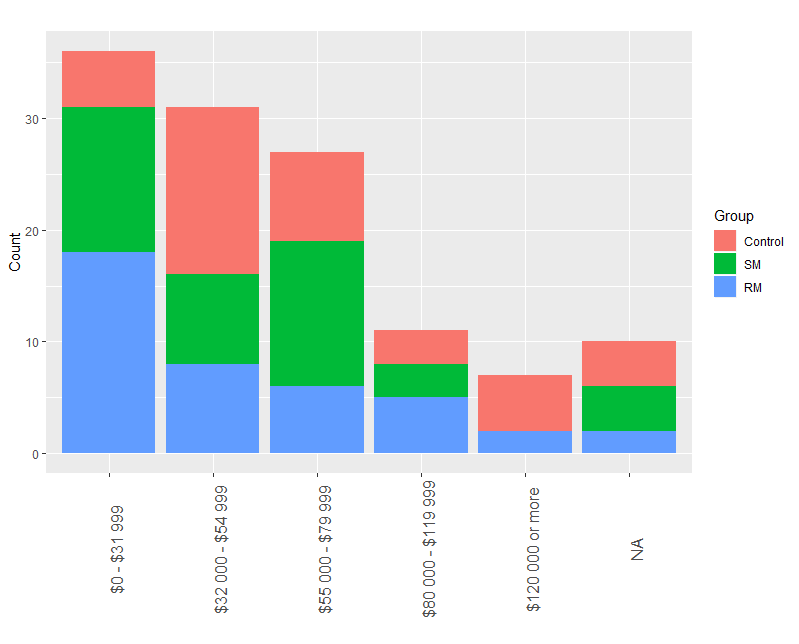


Figure 5. Rates of medical conditions among all participants.


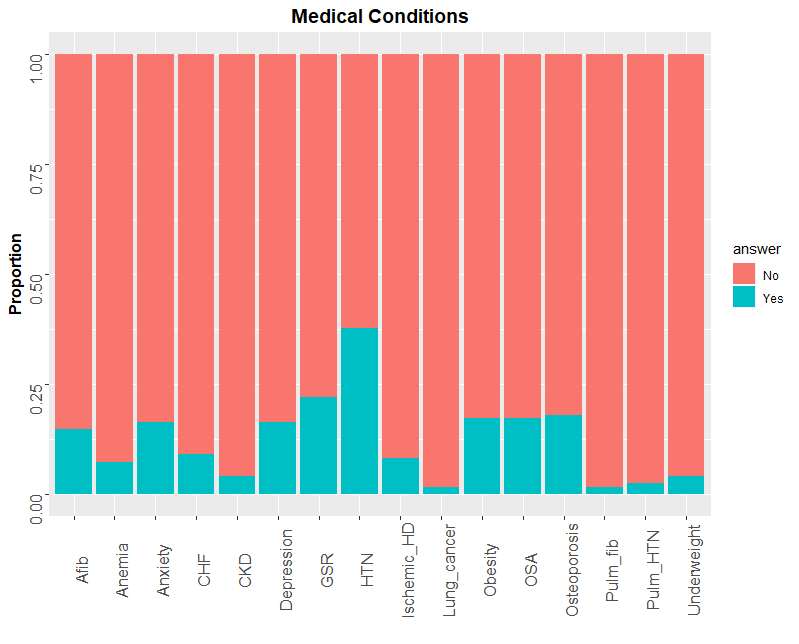


Figure 6: BCKQ at baseline and 3 months and baseline and 6 months for each group.


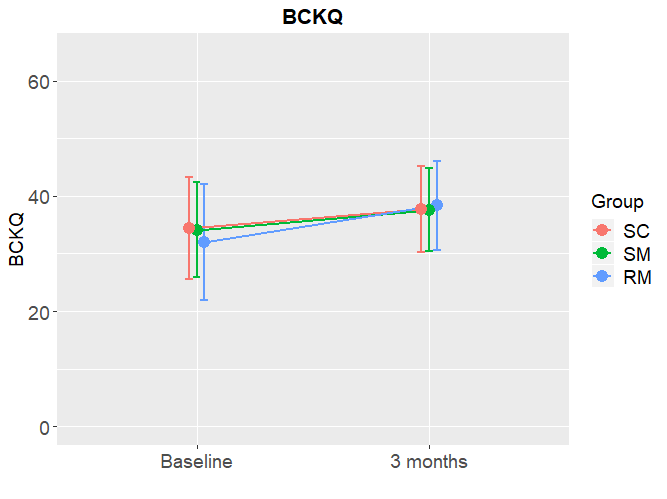

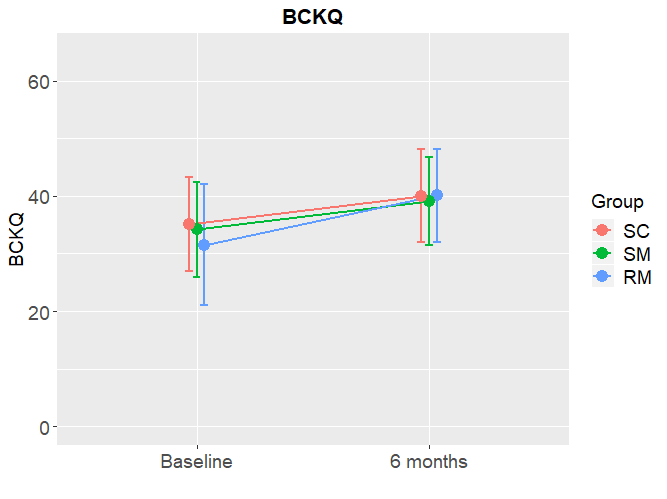


Figure 7: SGRQ Activity at baseline and 3 months and baseline and 6 months for each group.


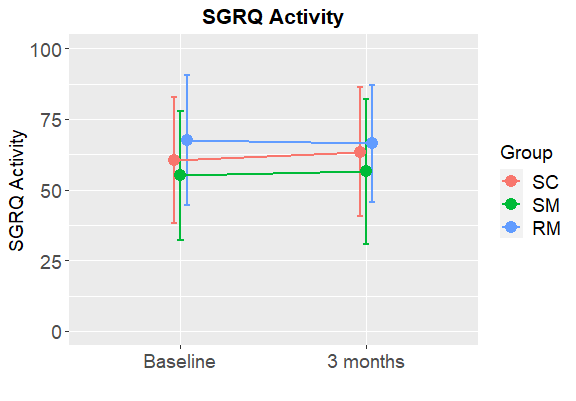

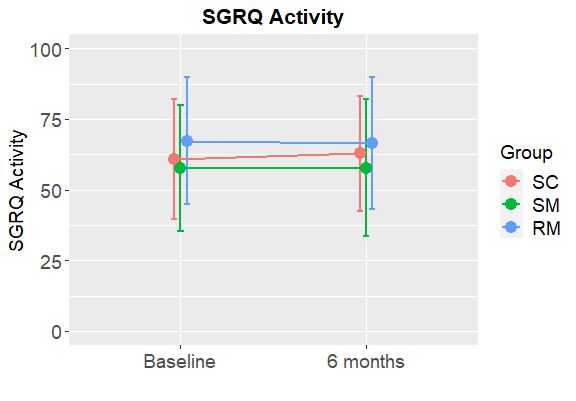


Figure 8: SGRQ Impact at baseline and 3 months and baseline and 6 months for each group.


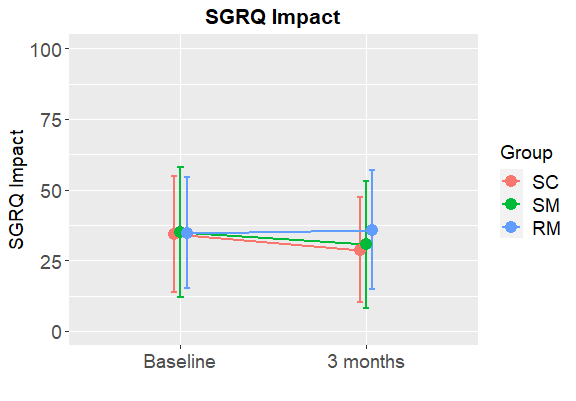

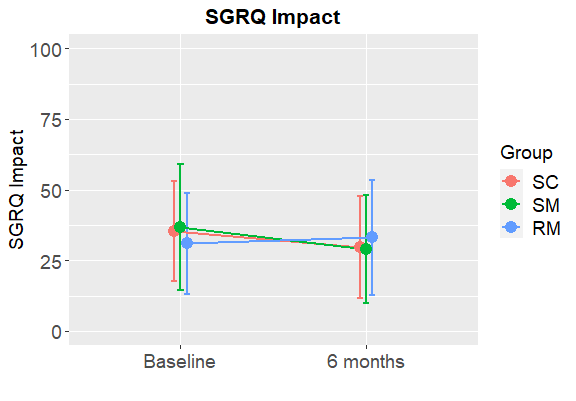


Figure 9: SGRQ Symptoms at baseline and 3 months and baseline and 6 months for each group.


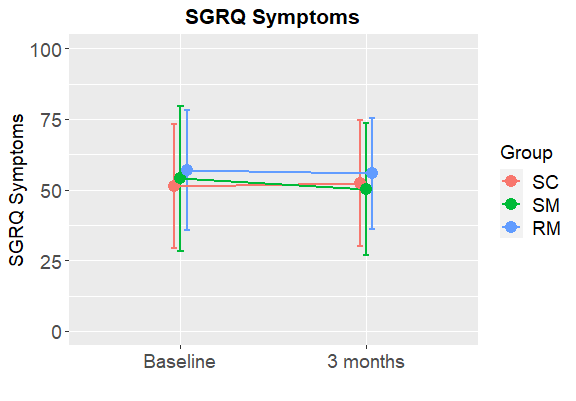

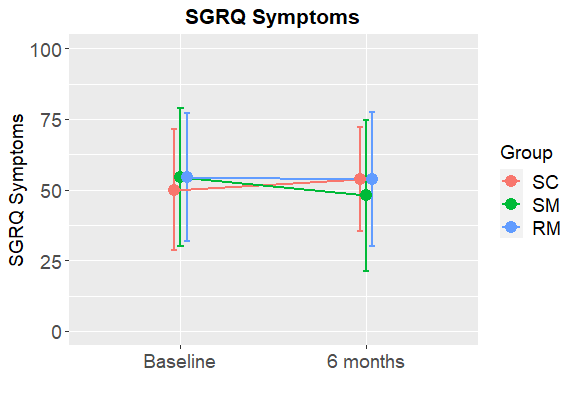


Table 1. Completed Readings and Notifications Sent per Group.

|  | **Type** | **Group** | **Median** | **mad** | **H** | **p-value** |
| --- | --- | --- | --- | --- | --- | --- |
| **Readings** | **MRC** | SM | 159.5 | 31.13 | 0.06 | 0.81 |
|  |  | RM | 161.5 | 31.13 |  |  |
|  | **CAT** | SM | 159 | 29.7 | 0.003 | 0.96 |
|  |  | RM | 157.5 | 30.4 |  |  |
|  | **SpO2** | SM | 251.5 | 120.1 | 0.045 | 0.83 |
|  |  | RM | 253 | 100.8 |  |  |
|  | **BP** | SM | 169 | 53.4 | 0.43 | 0.51 |
|  |  | RM | 184 | 55.6 |  |  |
|  | **Temperature** | SM | 8.5 | 11.12 | 1.21 | 0.27 |
|  |  | RM | 25 | 35.58 |  |  |
|  | **Weight** | SM | 39.5 | 57.08 | 0.18 | 0.67 |
|  |  | RM | 70.5 | 93.40 |  |  |
| **Notifications** | **MRC** | SM | 2 | 2.965 | 0.721 | 0.40 |
|  |  | RM | 3 | 4.448 |  |  |
|  | **CAT** | SM | 2 | 2.965 | 2.62 | 0.11 |
|  |  | RM | 5 | 4.448 |  |  |
|  | **SpO2** | SM | 3 | 4.448 | 0.314 | 0.58 |
|  |  | RM | 3 | 4.448 |  |  |
|  | **BP** | SM | 0 | 0 | 0.159 | 0.69 |
|  |  | RM | 0 | 0 |  |  |

Table 2. Patient-reported healthcare utilization

|  | | Baseline | | | | | | | | 3 months | | | | | | | | | | | | 6 months | | | | | | | | | | | | | | |
| --- | --- | --- | --- | --- | --- | --- | --- | --- | --- | --- | --- | --- | --- | --- | --- | --- | --- | --- | --- | --- | --- | --- | --- | --- | --- | --- | --- | --- | --- | --- | --- | --- | --- | --- | --- | --- |
|  |  | **n** | **mean** | **sd** | **median** | **mad** | **min** | **max** | **n** | | **mean** | **sd** | **median** | | **mad** | | **min** | | **max** | | **n** | | | **mean** | | **sd** | | **median** | | **mad** | | **min** | | **max** | |  |
| **EDA** | **Control** | 40 | 0.25 | 0.54 | 0 | 0 | 0 | 2 | 35 | | 0.06 | 0.24 | 0 | | 0 | | 0 | | 1 | | 33 | | | 0.15 | | 0.51 | | 0 | | 0 | | 0 | | 2 | |  |
|  | **SM** | 41 | 0.24 | 0.66 | 0 | 0 | 0 | 3 | 38 | | 0.34 | 0.78 | 0 | | 0 | | 0 | | 3 | | 36 | | | 0.39 | | 1.55 | | 0 | | 0 | | 0 | | 9 | |  |
|  | **RM** | 41 | 0.46 | 0.98 | 0 | 0 | 0 | 4 | 39 | | 0.15 | 0.37 | 0 | | 0 | | 0 | | 1 | | 34 | | | 0.18 | | 0.46 | | 0 | | 0 | | 0 | | 2 | |  |
|  |  | **n** | **mean** | **sd** | **median** | **mad** | **min** | **max** | **n** | | **mean** | **sd** | **median** | **mad** | | **min** | | **max** | | **n** | | | **mean** | | **sd** | | **median** | | **mad** | | **min** | | **max** | |  |  |
| **HA** | **Control** | 40 | 0.10 | 0.30 | 0 | 0 | 0 | 1 | 35 | | 0.03 | 0.17 | 0 | 0 | | 0 | | 1 | | 33 | | | 0.12 | | 0.42 | | 0 | | 0 | | 0 | | 2 | |  |  |
|  | **SM** | 40 | 0.10 | 0.38 | 0 | 0 | 0 | 2 | 38 | | 0.18 | 0.56 | 0 | 0 | | 0 | | 2 | | 36 | | | 0.06 | | 0.33 | | 0 | | 0 | | 0 | | 2 | |  |  |
|  | **RM** | 41 | 0.24 | 0.73 | 0 | 0 | 0 | 4 | 39 | | 0.51 | 2.57 | 0 | 0 | | 0 | | 16 | | 35 | | | 0.06 | | 0.24 | | 0 | | 0 | | 0 | | 1 | |  |  |
|  |  | **n** | **mean** | **sd** | **median** | **mad** | **min** | **max** | **n** | | **mean** | **sd** | **median** | **mad** | | **min** | | **max** | | **n** | | | **mean** | | **sd** | | **median** | | **mad** | | **min** | | **max** | |  |  |
| **Days in Hospital** | **Control** | 40 | 1.60 | 6.05 | 0 | 0 | 0 | 32 | 35 | | 0.03 | 0.17 | 0 | 0 | | 0 | | 1 | | 33 | | | 0.64 | | 2.55 | | 0 | | 0 | | 0 | | 14 | |  |  |
|  | **SM** | 40 | 0.85 | 4.35 | 0 | 0 | 0 | 27 | 38 | | 0.79 | 4.87 | 0 | 0 | | 0 | | 30 | | 36 | | | 0.28 | | 1.67 | | 0 | | 0 | | 0 | | 10 | |  |  |
|  | **RM** | 41 | 1.51 | 5.24 | 0 | 0 | 0 | 30 | 39 | | 0.74 | 2.57 | 0 | 0 | | 0 | | 14 | | 35 | | | 0.29 | | 1.18 | | 0 | | 0 | | 0 | | 5 | |  |  |
|  |  | **n** | **mean** | **sd** | **median** | **mad** | **min** | **max** | **n** | | **mean** | **sd** | **median** | **mad** | | **min** | | **max** | | **n** | | | **mean** | | **sd** | | **median** | | **mad** | | **min** | | **max** | |  |  |
| **Primary Care Any Reason** | **Control** | 40 | 1.10 | 1.22 | 1 | 1.48 | 0 | 5 | 35 | | 1.37 | 1.24 | 1 | 1.48 | | 0 | | 6 | | 33 | | | 1.48 | | 1.46 | | 1 | | 1.48 | | 0 | | 6 | |  |  |
|  | **SM** | 41 | 1.32 | 1.47 | 1 | 1.48 | 0 | 5 | 37 | | 1.51 | 1.57 | 1 | 1.48 | | 0 | | 6 | | 36 | | | 1.67 | | 1.60 | | 1 | | 1.48 | | 0 | | 6 | |  |  |
|  | **RM** | 41 | 1.46 | 1.50 | 1 | 1.48 | 0 | 7 | 39 | | 1.67 | 1.80 | 1 | 1.48 | | 0 | | 10 | | 35 | | | 1.66 | | 1.24 | | 1 | | 1.48 | | 0 | | 4 | |  |  |
|  |  | **n** | **mean** | **sd** | **median** | **mad** | **min** | **max** | **n** | | **mean** | **sd** | **median** | **mad** | | **min** | | **max** | | **n** | | | **mean** | | **sd** | | **median** | | **mad** | | **min** | | **max** | |  |  |
| **Primary Care COPD Related** | **Control** | 40 | 0.55 | 0.81 | 0 | 0 | 0 | 3 | 35 | | 0.31 | 0.53 | 0 | 0 | | 0 | | 2 | | 33 | | | 0.36 | | 0.60 | | 0 | | 0 | | 0 | | 2 | |  |  |
|  | **SM** | 41 | 0.59 | 0.92 | 0 | 0 | 0 | 3 | 39 | | 0.59 | 1.09 | 0 | 0 | | 0 | | 5 | | 36 | | | 0.42 | | 0.77 | | 0 | | 0 | | 0 | | 3 | |  |  |
|  | **RM** | 41 | 0.88 | 1.21 | 0 | 0 | 0 | 5 | 39 | | 0.44 | 0.79 | 0 | 0 | | 0 | | 3 | | 35 | | | 0.71 | | 1.07 | | 0 | | 0 | | 0 | | 4 | |  |  |
|  |  | **n** | **mean** | **sd** | **median** | **mad** | **min** | **max** | **n** | | **mean** | **sd** | **median** | **mad** | | **min** | | **max** | | **n** | | | **mean** | | **sd** | | **median** | | **mad** | | **min** | | **max** | |  |  |
| **Nursing Visits** | **Control** | 38 | 0.74 | 3.90 | 0 | 0 | 0 | 24 | 30 | | 0.07 | 0.25 | 0 | 0 | | 0 | | 1 | | 33 | | | 0.94 | | 4.18 | | 0 | | 0 | | 0 | | 24 | |  |  |
|  | **SM** | 40 | 0.08 | 0.35 | 0 | 0 | 0 | 2 | 37 | | 0.49 | 2.06 | 0 | 0 | | 0 | | 12 | | 34 | | | 0.18 | | 0.63 | | 0 | | 0 | | 0 | | 3 | |  |  |
|  | **RM** | 38 | 0.53 | 1.89 | 0 | 0 | 0 | 10 | 38 | | 0.32 | 1.23 | 0 | 0 | | 0 | | 7 | | 34 | | | 0.47 | | 1.52 | | 0 | | 0 | | 0 | | 8 | |  |  |
|  |  | **n** | **mean** | **sd** | **median** | **mad** | **min** | **max** | **n** | | **mean** | **sd** | **median** | **mad** | | **min** | | **max** | | **n** | | | **mean** | | **sd** | | **median** | | **mad** | | **min** | | **max** | |  |  |
| **Exacer-**  **bations** | **Control** | 40 | 0.68 | 1.14 | 0 | 0 | 0 | 6 | 35 | | 0.40 | 0.69 | 0 | 0 | | 0 | | 3 | | 33 | | | 0.48 | | 0.76 | | 0 | | 0 | | 0 | | 3 | |  |  |
|  | **SM** | 41 | 0.73 | 1.34 | 0 | 0 | 0 | 6 | 37 | | 0.73 | 1.41 | 0 | 0 | | 0 | | 7 | | 36 | | | 0.31 | | 0.62 | | 0 | | 0 | | 0 | | 2 | |  |  |
|  | **RM** | 41 | 0.76 | 1.22 | 0 | 0 | 0 | 5 | 39 | | 0.41 | 0.64 | 0 | 0 | | 0 | | 2 | | 35 | | | 0.80 | | 1.13 | | 0 | | 0 | | 0 | | 4 | |  |  |

EDA=ED Admissions, HA= Hospital Admissi
