# Supplementary material for: Technology-Enabled Self-Management of Chronic Obstructive Pulmonary Disease With or Without Asynchronous Remote Monitoring: Randomized Controlled Trial
Source: J Med Internet Res. 2020 Jul 30;22(7):e18598. doi: 10.2196/18598 (PMC7426797; doi:10.2196/18598)
Supplement: Multimedia Appendix 2 [file jmir_v22i7e18598_app2.docx]

Release Notes from your trial period:

Connected Health Platform

=========================

Feb 2019 - Web Release 3.11

  - Time tracking

  - Unified report

  - Scheduled surveys

Jan 2019 - Web Release 3.10.1

  - Service pack with bug fixes

Sep 2018 - Web Release 3.10

  - Initial launch of the Clinical Blood Pressure (CBP) metric

May 2018 - Web Release 3.9

  - Shared access for multiple clinicians and care providers

  - CAT/MRC score implementation and thresholds

  - Custom notifications templates

  - Additional notification functionality for temperature

  - Calendar integration

Connected Health Android

========================

Jan 2019 - Android Release 1.9.10121

  - Service pack with bug fixes

Jan 2019 - Android Release 1.9.10107

  - Hotfix with bug fixes

Dec 2018 - Android Release 1.9.11214

  - Hotfix with bug fixes

Dec 2018 - Android Release 1.9

  - Offline readings improvements

Nov 2018 - Android Release 1.8.11127

  - Hotfix with bug fixes

Sep 2018 - Android Release 1.8.11023

  - Hotfix with bug fixes

Sep 2018 - Android Release 1.8.10921

  - Hotfix with bug fixes

Sep 2018 - Android Release 1.8

  - Initial launch of the Clinical Blood Pressure (CBP) metric
